# To Understand the Elusive: How to Avoid the Disappearance of the Black Grouse at the Edge of Its Continuous Range?

**DOI:** 10.1002/ece3.71231

**Published:** 2025-04-23

**Authors:** Michał Adamowicz, Tomasz Gortat, Patryk Czortek, Michał Chiliński

**Affiliations:** ^1^ Department of Ecology and Animal Evolution, Institute of Ecology, Faculty of Biology University of Warsaw Warsaw Poland; ^2^ Białowieża Geobotanical Station, Faculty of Biology University of Warsaw Białowieża Poland; ^3^ Faculty of Biology, Imaging Laboratory University of Warsaw Warsaw Poland

**Keywords:** endangered species, environmental factors, Galliformes, mountains, tourism pressure

## Abstract

Galliformes are one of the most rapidly declining groups of bird species in Europe. The black grouse belongs to a species closely related to the types of habitats that are disappearing due to environmental changes caused by man, the climate crisis, and an increase in the number of predator species. While the populations of this species in Northern and North‐Eastern Europe are still relatively stable, in Central and Western Europe the black grouse is declining very quickly. For example, in Poland, there has been an approximately 100‐fold decrease in its population over the last 50 years. However, there is a difference between the rate of decline in black grouse numbers in Central European lowlands and mountain refuges—for example, the Alps and the Carpathians. The European mountains, still offering habitats shaped by relatively severe climate, may soon be the only type of habitat for this species to survive in this part of the continent. Our study aimed to indicate the main environmental factors determining the occurrence of the species in a mountain refuge, on the southwestern border of this species' continuous range. Based on a comprehensive model containing data on land cover by vegetation, topography, and human disturbance, we assessed environmental factors that shape the probability of black grouse occurrence in one of its last refuges in Europe. Our results reveal a trend for black grouse to prefer habitats of an early succession stage, and those can only persist in specific climatic conditions, or thanks to active protection. Detailed knowledge of the habitat choice of an endangered species constitutes valuable data necessary to avoid the fragmentation of remaining patches of its habitat, to assess the state of the environment in times of climate crisis, and to protect its features that ensure and increase the survival of vulnerable species, such as black grouse.

## Graphical abstract

Our study aimed to indicate the main environmental factors determining the occurrence of a species in a mountain refuge, on the southwestern border of this species' continuous range. Based on a comprehensive model containing data on land cover by vegetation, topography, and human disturbance, we assessed environmental factors that shape the probability of black grouse occurrence in one of its last refuges in Europe. Our results reveal a trend for black grouse to prefer habitats of an early succession stage, and those can only persist in specific climatic conditions, or thanks to active protection.

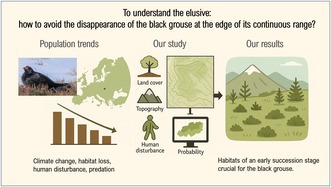

## 1 Introduction

Forest grouses are one of the most endangered groups of bird species in Europe. Populations of these species, for example, the black grouse ( *Lyrurus tetrix* ) in Fennoscandia, northern and northeastern Europe, are much more numerically stable than those in Central and Western Europe. In Finland, for example, there was a significant population decline starting in the 1960s, but the number of birds stabilized around 1980 (Jahren et al. 2016). The Polish black grouse population, which serves as a very good example of the rapidly dying out Central European population, has noted a constant 100‐fold decrease in numbers over the last 50 years (Polish Grouse Protection Committee meeting—Janów Lubelski 2023; Zawadzka 2014). Loss of habitat, increased predation pressure, human disturbance—especially tourism and winter sports, long‐term hunting, and the overall impact of climate change on the structure of environments are cited as the causes of this phenomenon (Jahren et al. 2016; Storch 2000; Zawadzka 2014), but there is still no clear answer as to which of them may be crucial for the survival of the disappearing local populations of this species. The black grouse has been described as an emblematic species of cold ecosystems that inhabits a broad range of boreal and mountainous habitats (Cramp and Simmons 1980) and the decrease of its population numbers has been described as slightly slower in mountainous habitats than in lowlands (Storch 2007). We assume that in the face of climate change, the mountains will become the main habitat type for this species to survive in this part of Europe.

Our study was conducted in the Tatra Mountains, which are one of the last refuges of this species in Central Europe. This small, dispersed but relatively stable population (Pęksa 2010) inhabiting an area on the fragmented border of the species' continuous range, and partially isolated, has so far been poorly researched. Studies on black grouse's environmental requirements show a positive impact of low vegetation land cover, for example, heather (Schweiger et al. 2012), and open forests with clearings, or meadows serving as lekking sites (Bernard‐Laurent 1994; Lindström et al. 1998; Magnani 1988). The affinities of the black grouse to specific types of habitats have been previously described and indicated the preferences of this species toward areas with an early stage of succession, heaths, peat bogs, and forest edges (Adamowicz 2019; Zawadzka 2014). Also, snow cover providing shelter in winter and wintering areas free from human disturbance are described as important for this species' occurrence (Keulen et al. 2003; Patthey et al. 2008). However, a study based on a comprehensive model containing information on the land cover by vegetation, landform, and human disturbance has not been carried out so far. Moreover, there is still not enough knowledge on factors shaping black grouse's occurrence at the border of its continuous range.

Our study aimed at finding habitats occupied by this species by conducting field inspections, and then, by statistical models, determining the main environmental factors necessary to preserve black grouse and defining key factors for the species' occurrence in a mountain refuge, on the southwestern border of this species' continuous range. It is also highly probable that the black grouse, as a species sensitive to environmental changes, can serve as an indicator of habitat quality for timberline ecosystems in alpine environments. This may be of particular importance both in relation to small isolated populations and populations inhabiting the borders of their global ranges, for which the fragmentation of patches is also a typical problem (Angelstam et al. 2000). The knowledge about environmental factors crucial for the survival of a declining population provides guidelines for managing areas to maintain the connectivity of appropriate habitats that meet its requirements. The black grouse's diet alone shows the importance of its habitat heterogeneity. It requires a diversity of plant species groups—for example, norway Spruce, european larch, arolla pine needles, heather, myrtle blueberry, crowberry, and bog bilberry leaves (Marti 1982), and for chicks—invertebrates living on bilberry shrubs and the bilberry shrubs themselves (Wegge and Kastdalen 2008).

As our hypothesis I, we predicted that mosaic habitats, composed of dwarf shrubs and forest edges, would be the most suitable for the black grouse. Concerning the abiotic factors, we expected the highest probability of black grouse occurrence above the upper tree line, in the mountain dwarf pine zone, on gently descending slopes, and with southern exposure (aspect), due to the increased sunlight, which supports better vegetation growth and provides food (hypothesis II). Also, the most favorable locations for this species would be those with a low amount of human disturbance, such as tourism and ski activity (hypothesis III).

Due to spatial isolation and the lack of external supply, our results may be relevant to other populations inhabiting areas at the boundaries of or beyond the species' continuous global range. Moreover, they may apply to populations within the continuous range. However, due to communication with other subpopulations and habitat continuity, this process may occur in different manners. Our results may be useful considering areas inhabited by other mountain species or boreal species, for which, in the face of climate change and environmental changes, mountains have remained the last refuge. They may also contribute to the protection of the black grouse, which entails the protection of the diversity of habitats located in the timberline zone, as research has shown that the heterogeneity of vegetation in the ecotone zones around the upper forest boundary is crucial for the occurrence of this species (Patthey et al. 2012). For the black grouse, our methods may be used as a tool for predicting the species occurrence. A model that incorporates biotic and abiotic parameters would allow for identifying suitable habitats for the black grouse, which would enable targeted site selection, verification of the birds' presence, and protection of those areas.

## 2 Study Area, Materials, and Methods

We chose the Tatra black grouse population as a model for assessing the habitat use of the black grouse at the edge of its continuous range. This area can serve as an example of patterns that can be observed on a wider spatial scale within black grouse sites in other European sites, especially in the mountains. The black grouse population in the Polish Tatra Mountains is part of the population inhabiting the entire Tatra Mountains, including the Slovak side. The Slovak Tatra Mountains are much larger than the Polish Tatra Mountains. The Slovak side is inhabited by 150–200 black grouse individuals, with a density of approximately 0.5 males per 100 ha (Mikoláš et al. 2018), and the population is described as slightly decreasing. The primary adverse factors affecting the Slovak population involve the expansion of open areas above the upper forest edge and the disturbances caused by sports activities, particularly skiing (Mikoláš et al. 2018). Certainly, groups of individuals residing in areas on either side of the border have direct interactions, and periodically, there is an exchange of individuals between them, as confirmed by our observations. Considering this and the similarity in geological structure (Dąbrowska and Guzik 2015), and human land use on both sides of the border, including national parks, designated tourist trails, and ski slopes, we assumed that the Polish black grouse population is a representative sample of the entire population of this species in the Tatra Mountains. Taking into account similar human use of land in other mountains—for tourism, winter sports, and locally grazing—we assume that the impact of these factors can be extrapolated to other mountain black grouse refuges in Europe.

We collected data on the occurrence of black grouse in the Tatra Mountains during two fieldwork seasons: 91 field inspections conducted systematically between 2020 and 2021, during which we recorded the geographic coordinates of each sighting, feather, droppings, or tracks found, or voice heard. For voice‐based records, we used the cross‐azimuth method (Chylarecki et al. 2015) which involves recording the azimuth of the direction from which the bird's voice comes from several locations while walking and then determining the bird's position at the intersection of these azimuth lines. All known black grouse sites in the Polish Tatra Mountains were visited by two to three observers (an ornithologist and field helpers) regularly and with a comparable number of field inspections in each of them from April 2020 to November 2021. For fieldwork, we selected days with good weather conditions, that is, no strong winds, prolonged precipitation, or extremely low temperatures. During field inspections, scheduled for the hours of black grouse activity, the observer walked along designated transects, with the length varying from 6,5 to 29 km, and recorded all signs of black grouse's presence. The differences in transect lengths were due to the mountainous terrain and the resulting varying accessibility of different mountain areas for observers. We designated the transects based on data from the Tatra National Park on current records of black grouse occurrence in the Polish Tatras, as well as historical data, and our observations, to cover all known sites occupied by the black grouse along the Polish Tatra Mountains. We designed the fieldwork plan to cover all black grouse's phenological periods (except for wintering—for fieldworker's safety) and to visit the same sites throughout the whole Polish Tatra Mountains in different seasons.

We noted 88 records of the black grouse: 54 based on found traces, 31 sightings, and three records based solely on voice heard. Although our black grouse records can also be divided into male records (41), female records (18), and tracks without the possibility of determining sex (29), we did not include this division in further analyses. The secretive behavior of females likely contributed to the lower number of female records. If we were to base our models solely on male records, we would have to exclude the remaining data, and such models would have low predictive power. We also do not have data on chicks and breeding success in the Tatra region. For these reasons, we decided to conduct further analyses based on all our recorded black grouse records.

**FIGURE 1:**
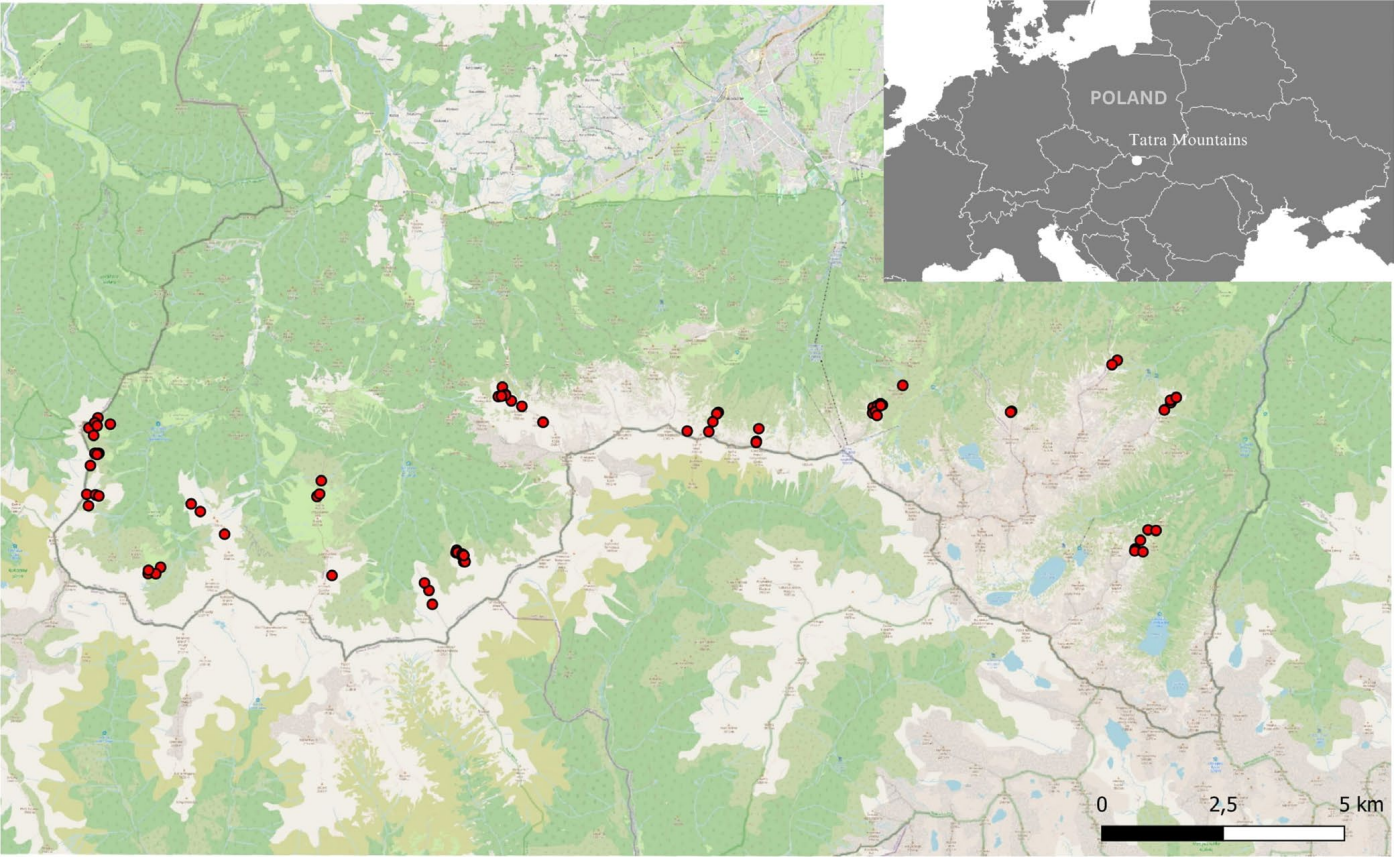
Distribution of black grouse records across the study area (with its location in a broader geographical context).

The records came from different seasons and from both years of fieldwork. Although we do not know the exact number of individuals from whom the records come it should be noted that they originate from the entire Polish Tatra region **(**Figure 1). Given the sedentary nature of the species (Borecha et al. 2017), and the fact that data on the occurrence of the black grouse from Tatra National Park overlaps with our locations, this provides a high likelihood of obtaining data from as many individuals as possible. Additionally, black grouse records come not only from the mating period (records noted in April, May, until mid‐June constitute only exactly 50% of all records) and not only from leks (which do not give complete knowledge about the type of habitat used by the birds) but also cover the sites of birds' occurrence in different phenological periods and seasons. The locations of our black grouse records align with the occurrence of the species as presented in the study by Santorek et al. (2021), conducted in this area and based on genetic material of the birds collected from all locations where the black grouse occurred then in the Polish Tatra Mountains. Therefore, we assume that the amount of terrain that we covered in the fieldwork was representative.

We performed the analysis of black grouse locations using the most popular open‐source GIS software (QGIS 3.24.2 Desktop version) and based on three sources: (a) Land cover classification from Kluczek et al. (2022) (13 classes, supervised classification, with accuracies for individual classes ranging from 76% to 90%); (b) digital Elevation Model (DEM) merged from two sources: Shuttle Radar Topography Mission and Polish nationwide LIDAR scanning mission (ISOK project) and (c) tourism pressure derived from traces of users of the Strava mobile application (source: freemap.sk). We used those three sources to analyze the relationships between black grouse occurrence and, respectively: land cover, altitude, and human disturbance. The years in which the field studies were conducted, despite the COVID‐19 pandemic, were marked by some of the highest numbers of visitors to the Tatra Mountains in history. In 2020, Tatrzański National Park sold 3,301,895 entrance tickets, and in 2021, 4,600,025 (data obtained from https://tpn.gov.pl/statystyki). Due to the lack of data on the number of people visiting black grouse habitats, as well as the absence of other available data used to measure human disturbance (e.g., the number of skier tracks on snow), we used data from Strava app users (Figure A1). While these data certainly do not provide a complete picture of tourist traffic in the Tatras, they may serve as a relatively reliable source of information on the distribution and intensity of human disturbance in the study area, given a large number of users (over 70 million users in 2020—source: *Strava releases 2020 Year In Sport Data Report* ). Strava tourism data are available in winter and year‐round categories. Tourism undoubtedly affects different aspects of black grouse biology depending on the season—during the skiing season, black grouse resting in snow burrows are at risk of disturbance, while in early spring, displaying males may be affected. In summer, on the other hand, hiking tourism can disturb females with chicks (Arlettaz et al. 2013; Patthey et al. 2012). However, since our models are based on all black grouse records (without distinguishing between sexes), we consistently apply the same approach to tourism data. For this reason, further analyses are based on Strava data from year‐round tourist activity.

To describe numerically habitat composition in the surrounding of observation points we used land cover classes (a). For the final model, we used only six out of the 13 land cover classes, which included: (i) coniferous forests (forest vegetation representing the *Piceion abietis* alliance with *Picea abies* composing the tree layer), (ii) dwarf pine communities (patches of *pinetum mugo* community with shrub layer composed by *Pinus mugo* subsp. *mugo*), (iii) dwarf shrub communities (representing the patches of *Empetro‐Vaccinietum* and *Vaccinietum myrtilli* communities), (iv) granite grasslands (representing the *Oreochloo distichae‐Juncetum trifidi*, *Calamagrostion*, and *Hieracio‐Nardetum* communities), (v) scree vegetation (communities representing the *Thlaspietea rotundifolii* class), and (vi) other vegetation (mosaics of low‐size patches of plant communities representing, for instance, hygrophilous tall herbs of *Adenostylion alliariae* alliance, and peatlands vegetation of *Oxycocco‐Sphagnetea* class). This generalization was due to the fact that classes such as rocks, shadows, snags, water, *Deschampsia flexuosa* tussocks, calcareous grasslands representing the *Festuco versicoloris‐Agrostietum* community, and snowbeds representing the *Luzuletum alpino‐pilosae* community had minimal representation in the field. We determined vertical boundaries of the extent of observations based on DEM approach (b). Additionally, we employed the process of raster analysis to derive slope and aspect values for observation points. As the Strava human disturbance data are a cumulative sum of information on tourist traffic, due to the lack of precise information (individual traces of skiers or hikers), we derived the tourism pressure (c) based on a simple binary approach, where all pixels with visible tracer were treated as pixel with pressure, while pixels without any trace were marked as “no pressure” areas.

We programmed the whole geoprocessing workflow with QGIS model builder, which allows easy recalculations and changes of input datasets (in case of new observations). Then, using QGIS software, we generated 88 random locations across the Polish Tatra Mountains, within the vertical range of this species in the Tatra Mountains. We took the lowest and the highest record (respectively 1503 m.a.s.l. and 1869 m.a.s.l.) as the limits of the vertical range based on the highest and lowest record noted during fieldwork, as well as based on data from the Tatra National Park (Pęksa 2010; Santorek et al. 2021) on the occurrence of this species in the Tatra Mountains. It is worth noting that although field inspections covered mainly the area above the upper forest edge, the access routes to these areas obviously led through lower vegetation zones, and no presence of black grouse was found there.

To determine the black grouse's habitat preferences, we compared land cover, topography, and human disturbance data from 88 black grouse records with the same data from 88 randomly drawn locations, which were generated within the vertical range of the black grouse's occurrence in the Tatra Mountains, using the QGiS software. The aim was to determine whether the black grouse selects locations randomly or if there are preferences toward specific biotic and abiotic habitat parameters. We have not confirmed the black grouse presence in those randomly generated locations, but potentially they could also be visited by the birds. Those random locations are further in this paper defined as pseudoabsences. To avoid distorted results due to the small sample size, we performed 10 draws of pseudoabsence locations. This number of draws was also performed to cover the entire area of black grouse occurrence across the Polish Tatra Mountains.

### 2.1 Data Analysis

All statistical analyses were conducted using R (version: 4.3.1; R Core Team 2023). A comparison of the affinity of pixels representing black grouse's pseudoabsences to land cover classes revealed relatively large differences among ten draws (Figure A2a). For instance, the number of pixels representing dwarf shrub communities ranged from 17 to 45, while the number of pixels classified as dwarf pine communities ranged from 11 to 27. Smaller, but noticeable differences among ten draws we identified in regards to the classification of black grouse's pseudoabsences to tourism presence (Figure A2b). The number of pixels without tourism ranged from 67 to 77 among draws, and the number of pixels with tourism ranged from 11 to 21.

Using linear models (LMs) evaluated by ANOVA (*base::aov()* function), we checked whether there were differences in mean elevation, aspect, and slope among ten draws. That allowed us to capture and compare the variation of those factors in the study area, and to confirm the correctness of the draw, and to provide representative sets of black grouse pseudoabsences for the analyzed characteristics. For the global model, we analyzed variance inflation factors and, in each case, we did not detect multicollinearity among the predictors. This was reflected in VIF values < 5 for each predictor. While visualizing the results of LMs, we accounted for marginal responses, and we used Tukey posteriori tests with studentized adjustment for multiple hypothesis testing (*emmeans::emmeans()* function (Lenth 2023). We found that the differences in mean elevation above sea level, aspect, and slope among ten draws of 88 black grouse's pseudoabsences were not statistically significant (Figure A3a–c and Table A3; Appendix S1). Differences in mean elevation above sea level among ten draws were slight (Figure A3a; Appendix S1). We identified the draw no. 10 as having the highest mean aspect (209^o^), while draws no. 7 and no. 5 were characterized by the smallest values of this parameter (of 44^o^ and 36^o^) smaller than regarding the draw no. 10, respectively; (Figure A3b and Appendix S1). We found that draw no. 4 had the highest mean slope (28.70^o^), whereas draw no. 3 exhibited the lowest values for this parameter (3.70^o^) lower than draw no. 4; (Figure A3c and Appendix S1). The mean values for altitude, slope, and aspect for 10 draws of pseudoabsences are presented in Table A2 (Appendix S1).

Building upon the patterns described above, we discovered relatively distinct differences in the variability of some of the environmental characteristics of the pixels used for the conductance of ten draws of 88 black grouse's pseudoabsence localities in the study site. This enabled us to consider that the process of selection of pixels representing the pseudoabsences of black grouse, which potentially would be part of the set of 88 pixels used for the subsequent draw, was crucial in formulating an assessment regarding the environmental characteristics of locations where black grouse was potentially absent. Therefore, to test the effects of environmental characteristics on the probability of this bird species occurrence, we built a set of ten initial generalized linear models (GLMs) assuming the binomial distribution of the response variable. Each initial GLM consisted of a set of all hypothesized predictors (i.e., land cover class, tourism presence, elevation above sea level, aspect, and slope). The portions of each predictor's value ranges representing the pseudoabsences of black grouse within the study area were determined through the execution of each of ten draws. To select the global GLM, which best predicted the probability of the black grouse occurrence, we employed the corrected Akaike information criterion (AICc), implemented in the *MuMin::AICc()* function (Bartoń 2023). On this basis, we selected a GLM with the lowest AICc value as a full model for further analyses. Then, we reduced the full model to minimize AICc, using the *MuMIn::dredge()* function (Bartoń 2023), assuming that GLMs differing in AICc by < 2 have equivalent predictive power in explaining the occurrence patterns of black grouse. To depict differences in the probability of black grouse occurrence among six land cover types and under an impact of tourism, we utilized marginal responses and conducted Tukey post hoc tests, using the *emmeans::emmeans()* function (Lenth 2023). To illustrate the influence of elevation above sea level, aspect, and slope on the probability of black grouse occurrence, we employed the *ggeffects::ggpredict()* function (Lüdecke 2023). Implementing this approach allowed us to assess the effect sizes of the observed patterns, shifting the focus from the mere statistical significance of the results obtained to their biological importance (Nakagawa and Cuthill 2007; Wasserstein and Lazar 2016).

## 3 Results

**FIGURE 2:**
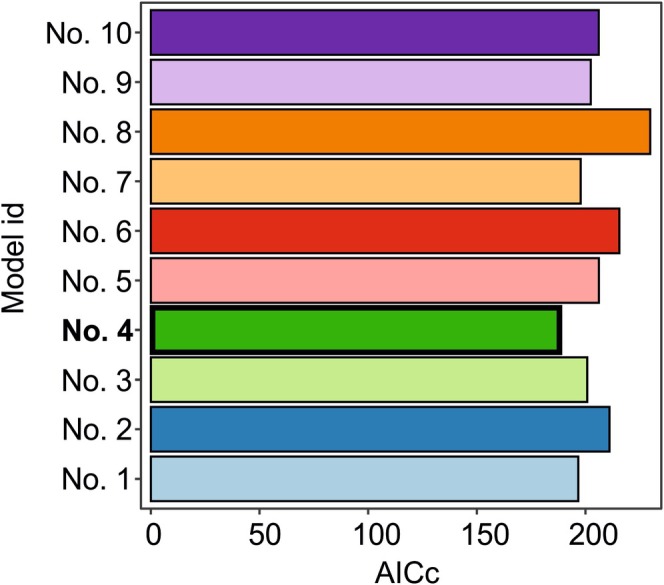
A comparison of AICc values of ten initial GLMs differing in terms of the predictors' value ranges representing the pseudoabsences of black grouse, determined through the execution of each of 10 draws. Black frame and bolded text denote the initial model with the lowest AICc, which was adopted as the global model for further analyses (model no. 4).

**FIGURE 3:**
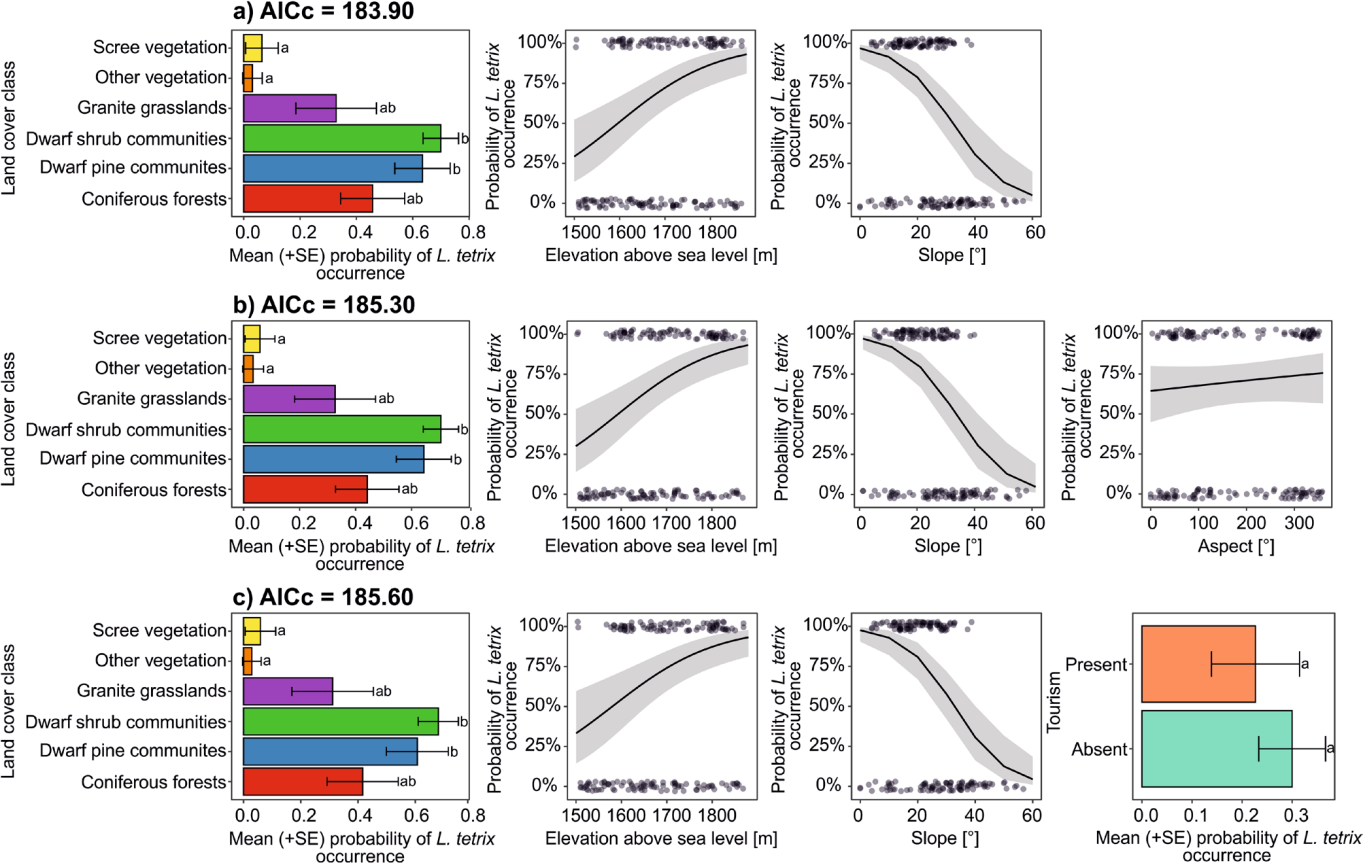
Visualization of three final generalized linear models equally best predicting (included in a set of GLMs with ΔAICc < 2) the probability of occurrence of black grouse depending on land cover, elevation above sea level, slope, aspect, and tourism pressure. Comparisons of the importance of land cover classes and tourism pressure were based on Tukey's posteriori tests. Letters show significance of results from the post hoc Tukey's posteriori test. Groups marked by the same letter do not differ significantly at *p* = 0.05. For model parameters see Table 1.

Comparing the AICc values of ten initial GLMs, which differed in regard to the predictors' value ranges representing the pseudoabsences of black grouse, determined through the execution of each of 10 draws, (and also taking into account that the differences between the individual draws were not statistically significant), we chose **model no. 4** (built based on results of draw no. 4) as a full GLM the best predicting the occurrence patterns of black grouse in the study area (Figure 2). Based on the results of the model selection procedure, we identified three GLMs as equivalent (differing in AICc by < 2) in explaining the probability of the black grouse occurrence (Table A1). The model with ΔAICc = 62.11 (in relation to intercept‐only model with AICc = 227.40) contained three predictors: land cover class, elevation above sea level, and slope (Figure 3a). Aside from the three predictors present in the previous GLM, the model with ΔAICc = 60.71 (in relation to intercept‐only model) contained aspect as an additional predictor (Figure 3b), and the model with ΔAICc = 60.41 (in relation to intercept‐only model) contained the presence of tourism as another explanatory variable (Figure 3c).

Based on the visualization of all three models, we identified similar effect sizes of land cover class, elevation above sea level, and slope on the probability of black grouse occurrence (Figure 3a,c and Table 1). We found that the probability of this bird species' presence was the highest in localities covered by dwarf shrub and dwarf pine vegetation (68%–70% and 61%–64%, respectively), and was the lowest in pixels classified as scree vegetation and other plant communities (about 6% and 3%, respectively, in all three models). Regarding the localities representing coniferous forests and granite grasslands, the probability of black grouse occurrence was about 42%–46% and 31%–33%, respectively. The probability of black grouse presence increased with increasing elevation above sea level: from about 29%–33% at 1500 m to 93% at 1880 m above sea level, and decreased with increasing slope: from about 4%–5% at 60° to 97%–98% at 0°. Taking into account the results of GLM with ΔAICc = 60.71, the probability of black grouse occurrence increased with increasing aspect, from approximately 64% at 0° to 76% at 360°, which should be understood in terms of cardinal directions: the probability was lowest in northern locations and increased toward the east, south, and up to the northwest. Please provide the sentences you would like me to rewrite. (Figure 3b and Table 1). Considering the results of GLM with ΔAICc = 60.41, the probability of black grouse occurrence was about 8% lower in localities with the presence of tourism than in pixels where tourism pressure was absent (Figure 3c and Table 1).

**TABLE 1:** Parameters of three final generalized linear models (GLM) equally best predicting (with a difference in AICc < 2) the probability of occurrence of black grouse depending on land cover, elevation above sea level, slope, aspect, and tourism pressure.

Predictor	Estimate	SE	*z*	*p*
*GLM with ΔAICc = 62.11*:				
(Intercept)	−13.094	3.819	−3.429	**< 0.001**
Land cover class = dwarf pine communities	0.721	0.605	1.191	0.233
Land cover class = dwarf shrub communities	**1.014**	**0.536**	**1.891**	**0.058**
Land cover class = granite grasslands	−0.547	0.839	−0.652	0.514
Land cover class = other vegetation	−3.275	1.239	−2.643	**0.008**
Land cover class = scree vegetation	−2.503	1.100	−2.274	**0.022**
Elevation above sea level	0.009	0.002	3.841	**< 0.001**
Slope	−0.106	0.021	−4.947	**< 0.001**
*GLM with AICc = 60.71*:				
(Intercept)	−13.285	3.834	−3.465	**< 0.001**
Land cover class = dwarf pine communities	0.826	0.615	1.343	0.179
Land cover class = dwarf shrub communities	1.103	0.544	2.026	**0.042**
Land cover class = granite grasslands	−0.489	0.844	−0.579	0.562
Land cover class = other vegetation	−3.121	1.230	−2.537	**0.011**
Land cover class = scree vegetation	−2.545	1.096	−2.321	**0.020**
Elevation above sea level	0.009	0.002	3.815	**< 0.001**
Slope	−0.108	0.021	−4.971	**< 0.001**
Aspect	0.001	0.001	0.901	0.367
*GLM with AICc = 60.41*:				
(Intercept)	−12.183	4.008	−3.040	**0.002**
Land cover class = dwarf pine communities	0.774	0.609	1.271	0.203
Land cover class = dwarf shrub communities	1.095	0.547	2.000	**0.045**
Land cover class = granite grasslands	−0.455	0.854	−0.533	0.593
Land cover class = other vegetation	−3.176	1.241	−2.559	**0.010**
Land cover class = scree vegetation	−2.429	1.092	−2.223	**0.026**
Elevation above sea level	0.008	0.002	3.560	**< 0.001**
Slope	−0.112	0.023	−4.782	**< 0.001**
Tourism pressure = present	−0.379	0.521	−0.728	0.466

*Note:* AICc of null (intercept‐only) model = 227.40. ΔAICc refers to null (intercept‐only GLM). Statistically significant results are marked in bold.

## 4 Discussion

Our results confirmed the hypothesis I, presenting the black grouse as a species inhabiting a mosaic of open areas covered with dwarf shrubs and forest edges. Since our study did not show a dominance of southern slopes among the black grouse occurrence sites, we cannot consider hypothesis II as fully confirmed. However, our results did confirm the predicted vegetation zone preferred by black grouse and the slight slope gradient. Similarly, in the case of hypothesis III, our predictions regarding the avoidance of human disturbance were generally correct, but we could only fully confirm hypothesis III if there were a significant negative correlation between black grouse occurrence and this factor, which we did not find.

The black grouse biotope is usually a mosaic of open, semi‐open, and forest areas (Adamowicz 2019). The comparison of the land cover in locations of black grouse records with the land cover in the locations obtained in ten draws allows us to detect two clear preferences of this species for particular classes of land cover. Firstly, the results indicate the black grouse's preference for sites covered by dwarf shrubs. Also, in our model, the highest probability of occurrence of black grouse is in this land cover type. This type of land cover provides an open area—an inherent element of the diverse landscape of the upper forest border and its ecotone zone, and also an important component of the black grouse's biotope—the grouse use open areas as lekking sites in the mating season in spring and also for autumn leks (Adamowicz 2019). Moreover, the fruits of shrubs are an important element of the black grouse's food base (Beeston et al. 2005; Malkova 2000) during the breeding season. In the case of the Tatra Mountains, it is expected to be European blueberry ( *Vaccinium myrtillus* ), lingonberry ( *Vaccinium vitis‐idaea* ), crowberry ( *Empetrum nigrum* ) and common heather ( *Calluna vulgaris* ) (Radwańska‐Paryska 1988). Therefore, it is important to protect that land cover type in order to maintain the suitable habitat for black grouse. The second highest probability of occurrence of black grouse according to our results is in dwarf pine ( *Pinus mugo* ). This type of vegetation, together with the low vegetation of dwarf shrubs, ensures the mosaic landscape which is typical for the black grouse biotope, above the upper forest edge. Dwarf pine can also provide shelter for the black grouse. Although our data do not include any knowledge on this period, it is expected to be especially important during summer molting and chick‐rearing, which are periods of increased predator avoidance (Kamieniarz 2004; Siitari et al. 2007). The next type of land cover in the order of probability of occurrence of black grouse is coniferous forest. During fieldwork, we found the presence of black grouse in the range of 1503–1869 m above sea level. In the lower limit of the vertical range of the species, in most areas inhabited by the black grouse, there is a spruce forest getting thinner along with altitude. Forest areas are one of the three (next to open and semi‐open areas) important components of the black grouse biotope, primarily providing shelter. It is worth noting here, however, that succession resulting from the interaction of grazing cessation and climate change may be the most likely scenario in that area (Czortek et al. 2018a, 2018b, 2018c; Steinbauer et al. 2018). This process may lead to the competitive exclusion of open habitats by generalist plant species and the loss of open areas used by black grouse as lek sites and for foraging. Another study of habitat use by black grouse, based on using telemetry (Patthey et al. 2012) indicates that chick‐rearing females need very specific, heterogeneous habitats rich in invertebrates to feed their chicks. Perhaps it is the females who avoid human infrastructure—roads and trails—not only due to direct human disturbance but also their negative impact on the plant cover. A type of land cover also ensuring the open character of the area, apart from dwarf shrubs, was granite grasslands. However, it was not so strongly preferred by the black grouse as dwarf shrubs, and also it was only the fourth land cover class in terms of probability of black grouse occurrence. The other two types of land cover—other vegetation and rock screes were—the least frequently chosen by grouse and the probability of occurrence of these birds in them is low. Bare rocks do not provide food or lekking sites, but due to the lack of natural cover, it can expose birds to predator attacks; thus, avoiding this type of environment is understandable. As the other vegetation class is a class composed of unspecified groups of plant species, it is a negative preference we are unable to interpret.

The habitats occupied by black grouse in the studied area, consisting of dwarf shrubs, granite grasslands, scattered dwarf pine bushes, and the upper tree line, can certainly be described as being in an early stage of succession, which confirms our hypothesis (I). Also, research conducted on relatively numerous and stable populations, such as in Finland, indicates that black grouse is attached to early forms of succession. For example, the study by Hovi et al. (1996), (however based on lekking sites—which does not provide full knowledge about the use of the habitat) shows the preference by black grouse for bogs and peat bogs (and even frozen lake surfaces). Also, a negative relationship is found between mean tree age and the occupancy of black grouse in boreal forests (Mazziotta et al. 2024). A study by Wegge and Rolstad (2011) on black grouse from Fennoscandia focusing on the relationship between the occurrence of forest grouse and the stage of succession in the forests, again shows the black grouse's attachment to early stages. As the height of cover of plants increased, the number of birds in a given area decreased. At the same time, the authors indicated that in that population, changes in the predator regime are a more important factor than forest structure. Of course, we cannot rule out the importance of this factor in our study population, but our study did not include it.

Studies focusing on the habitat use of black grouse inhabiting areas outside the continuous global range—in this case, northern England (Starling‐Westerberg 2001)—indicate the birds' attachment to grassland habitats and heather moorlands. As suggested by these authors, the conservation tasks for this westernmost population include regulation of sheep‐grazing and mowing hay fields. Also, the results of a study by Czortek (2018) conducted in Tatra Mountains show that climate change leads to plant composition shifts, which easily can result in changes in black grouse's habitats. Succession, starting with dwarf pine, then carpathian mountain ash (*Sorbus carpatica*), and lastly Norway spruce (Czortek 2018), can cause limitation of open areas inhabited by the black grouse. This suggests the conclusion that outside its continuous range, the black grouse's biotope is often a transitional state. As climate change poses a major threat to wetlands (Salimi et al. 2021), we can expect further destabilization of those black grouse habitats, where open areas are associated with the presence of peatlands. The early‐successional habitat, rich in open areas and clearings, occurring in a stable state has a higher chance of survival in northern latitudes (northern Europe) or high altitudes (timberline ecosystems) determining the climate. Elsewhere, such conditions may also occur in natural areas where the dynamics of forest gaps (due to fires, invertebrate outbreaks, etc.) is undisturbed. In other cases, the black grouse habitat requires active protection or will disappear. It leads to the conclusion that the existence of the black grouse habitats at the edges, or outside of its continuous range, most likely requires active protection, aimed at controlling mainly the climate change‐caused factors such as succession. This means that its habitats cannot sustain themselves at a sufficient level without human attempts to reverse the negative effects of climate change. In this case, mountains such as the Tatra Mountains, where snowy winters still prevail and the upper forest boundary provides heterogeneity of low‐successional habitats, may still represent some of the best habitats for the survival of the black grouse at the boundaries of its continuous range.

The preferences of black grouse toward (I) locations of lower slope inclination than pseudoabsence locations and (II) locations of slightly greater height above sea level than random locations should be explained by the attachment of the grouse to leks and their vicinity. Black grouse leks take place in relatively flat open areas, and such conditions are met by the bottoms of valleys located above the upper limit of the forest, but especially (also confirmed by our own observations) on mountain ridges, peaks, and passes (Lindström et al. 1998; Pęksa 2010).

The alternative model taking into account the mean aspect of the locations of black grouse records indicates that the birds chose the northern side—the probability of occurrence increased along with an increasing aspect value. However, to correctly interpret this result, the distribution of aspect values for individual records should be taken into account. The averaged aspect value resulted from the dominance of the records located on the eastern and western sides of slopes descending from the north‐oriented ridges, perpendicular to the main Tatra ridge. They constituted over 75% of all our black grouse records. We conducted the research in the Polish part of the Tatra Mountains, that is, on the northern side of the main ridge. The main, east–west oriented ridge of the Tatra Mountains, due to its height, steep slopes, and poor vegetation, does not meet the habitat requirements of the black grouse. However, the lower, flattened ridges extending perpendicularly from the main ridge toward the north were suitable for settlement by the black grouse. Most of the records come from eastern and western slopes descending from those ridges. Aspect values slightly above zero (eastern slopes) and aspect values slightly below 360^o^ (western slopes) may represent different habitat conditions (primarily due to differences in sunlight exposure duration) where we recorded the presence of black grouse. The hypothesis II was confirmed partially. Southern slopes did not dominate in terms of the number of black grouse occurrences. However, we correctly predicted the occurrence of this species in locations with relatively low slope values and in the dwarf pine zone.

Galliformes, especially black grouse and capercaillie, are known to be anthropophobic species (Arlettaz et al. 2013; Tost et al. 2020). Most of the locations of black grouse records were noted in human disturbance‐free areas, and also the probability of black grouse occurrence was 8% lower in areas covered by human disturbance, which partially confirms our hypothesis (III). However, we did not find a significant negative correlation between locations preferred by black grouse and human disturbance coverage. Considering studies indicating elevated corticosterone levels in black grouse in areas with intensive sport, especially ski use (Arlettaz et al. 2013; Formenti et al. 2015), as well as studies showing the impact of frequent flushing on extending the distance for which these birds escape (Baines and Richardson 2007), we find it very likely that our study was unable to capture the actual impact of human disturbance on the grouse population, as this effect is not expressed in the distribution (black grouse are very attached to their habitats, especially lekking grounds (Borecha et al. 2017)), but rather in the individual condition of the birds and their activity. Since we do not have clear information on the impact of specific elements of human disturbance (winter sports, hikers), we cannot be certain about the causes of these results. However, we hypothesize that the lack of a significant negative correlation between tourism and black grouse occurrence might be due to the possibility of the presence of people resulting in scaring away the predators. On the other hand, extensive tourism pressure can also increase predation on birds due to habitat fragmentation (Storch et al. 2005), and also increase predator densities in black grouse refuges, as a consequence of providing food leftovers by tourists leaving garbage (Storch and Leidenberger 2003; Wittwer and Arlettaz 2007). Another alternative model we designed shows the probability of black grouse occurrence in different land cover types including human disturbance. Differences in the black grouse's preferences for land cover types differ only slightly while taking human disturbance into account. However, since we could not separate the results for males, females, and chicks due to fieldwork difficulties, secretive nature of the birds, and small population size, our results should be interpreted with caution in this regard. Although we cannot rule out the possibility of the future impact of extensive tourism on habitat quality, as mentioned, for example, in studies showing the negative impact of skiing homogenization of vegetation due to skiing on slopes (Patthey et al. 2012), our results show that attachment to certain land cover types is now stronger in black grouse than attachment to areas devoid of human disturbance. In this case, it would be assumed as the reason for the fact that there is no major difference between the probability of black grouse occurrence in disturbed and undisturbed areas. However, considering the overall negative impact of tourism on grouse populations as indicated in numerous studies, and also due to the dense network of tourist trails in most European mountains and the growing phenomenon of illegal off‐trail free‐riding, it is recommended to make tourist traffic maximally channelized to protect grouse species.

## 5 Conclusions

The results of all models clearly indicate that the key factors for the occurrence of the black grouse in the refuge located on the border of the continuous range of this species are the cover of the area with vegetation providing food and the open nature of the area, which is one of the main described elements of its biotope, consisting of a mosaic of open, semi‐open, and forested areas. Protection of such habitats clearly helps to maintain the heterogeneity of timberline habitats in mountainous environments. Since the black grouse is attached to habitats at an early stage of succession (e.g., dwarf shrubs), land management aimed at preventing succession should be considered as part of protection. Probably, as a consequence of the cessation of sheep grazing and as climate change progresses, we will see changes in the ranges of species from lower to higher locations (mainly of generalists with greater competitive abilities: dwarf pine, carpathian mountain ash, spruce) above all the upward shift of the vegetation zones, and therefore also with progressive succession in the black grouse habitats. It may turn out that to preserve vulnerable habitats such as timberline ecotones and the last, fragmented populations of species such as the black grouse, it will be necessary to change the development of some areas from tourism‐oriented to active protection.

Human disturbance seems to influence the distribution of this species to a lesser extent. However, it should be remembered that the impact of stress may be undetectable in our study and may concern the individual condition and reduced activity of birds inhabiting the studied areas. The results of these studies can be treated as an essential element—in addition to knowledge about predation pressure, individual condition, and breeding success—in assessing the preferences for environmental factors of the black grouse. Being able to precisely describe the selection of places that meet the requirements of this species, we will be able to identify places of occurrence of this species in other locations, as well as to protect this type of habitat in other refuges. This will help to avoid the increasing fragmentation of patches by ensuring the continuity of the suitable habitats that maintain the population.

## Author Contributions

**Michał Adamowicz:** conceptualization (lead), data curation (equal), formal analysis (supporting), investigation (equal), methodology (equal), project administration (lead), writing – original draft (lead). **Tomasz Gortat:** conceptualization (equal), data curation (equal), investigation (equal), methodology (equal), project administration (equal), supervision (lead), writing – original draft (supporting), writing – review and editing (lead). **Patryk Czortek:** conceptualization (supporting), data curation (supporting), formal analysis (lead), methodology (equal), software (lead), visualization (lead), writing – review and editing (equal). **Michał Chiliński:** conceptualization (supporting), data curation (equal), methodology (equal), software (equal), writing – review and editing (equal).

## Ethics Statement

The fieldwork was conducted under the permits from the management of Tatra National Park No. 238 from 16.12.2019 to 18.12.2020, and the decision of the Ministry of Climate and Environment no. 1240306.3851845.3102191.

## Conflicts of Interest

The authors declare no conflicts of interest.

## Pre‐Register

URL: https://osf.io/

## Supporting information


**FIGURE A1.** Distribution of tourist traffic in Polish Tatra Mountains (purple lines; source: Strava; freemap.sk), and black grouse occurrence (red points).

**FIGURE A2.** A comparison of the affinity of locations representing black grouse’s pseudoabsences to land cover classes (a) and tourism pressure (b) among ten draws of pixels representing pseudoabsences of black grouse.

**FIGURE A3** Differences in mean (+SE) elevation above sea level (a), aspect (b), and slope (c) among ten draws of pixels representing pseudoabsences of black grouse. Comparisons were based on ANOVAs and Tukey’s posteriori tests. Letters show the significance of results from the post hoc Tukey’s posteriori test. Groups marked by the same letter do not differ significantly at *p* = 0.05. For model parameters see Table A3.

Appendix S1.

## Data Availability

The data that support the findings has been provided for review process as supporting material and further it is available from the corresponding author upon reasonable request. Due to protection of an endangered species data is not public.
