# Supplementary figures and images for: To Understand the Elusive: How to Avoid the Disappearance of the Black Grouse at the Edge of Its Continuous Range?
Source: Ecol Evol. 2025 Apr 23;15(4):e71231. doi: 10.1002/ece3.71231 (PMC12015741; doi:10.1002/ece3.71231)

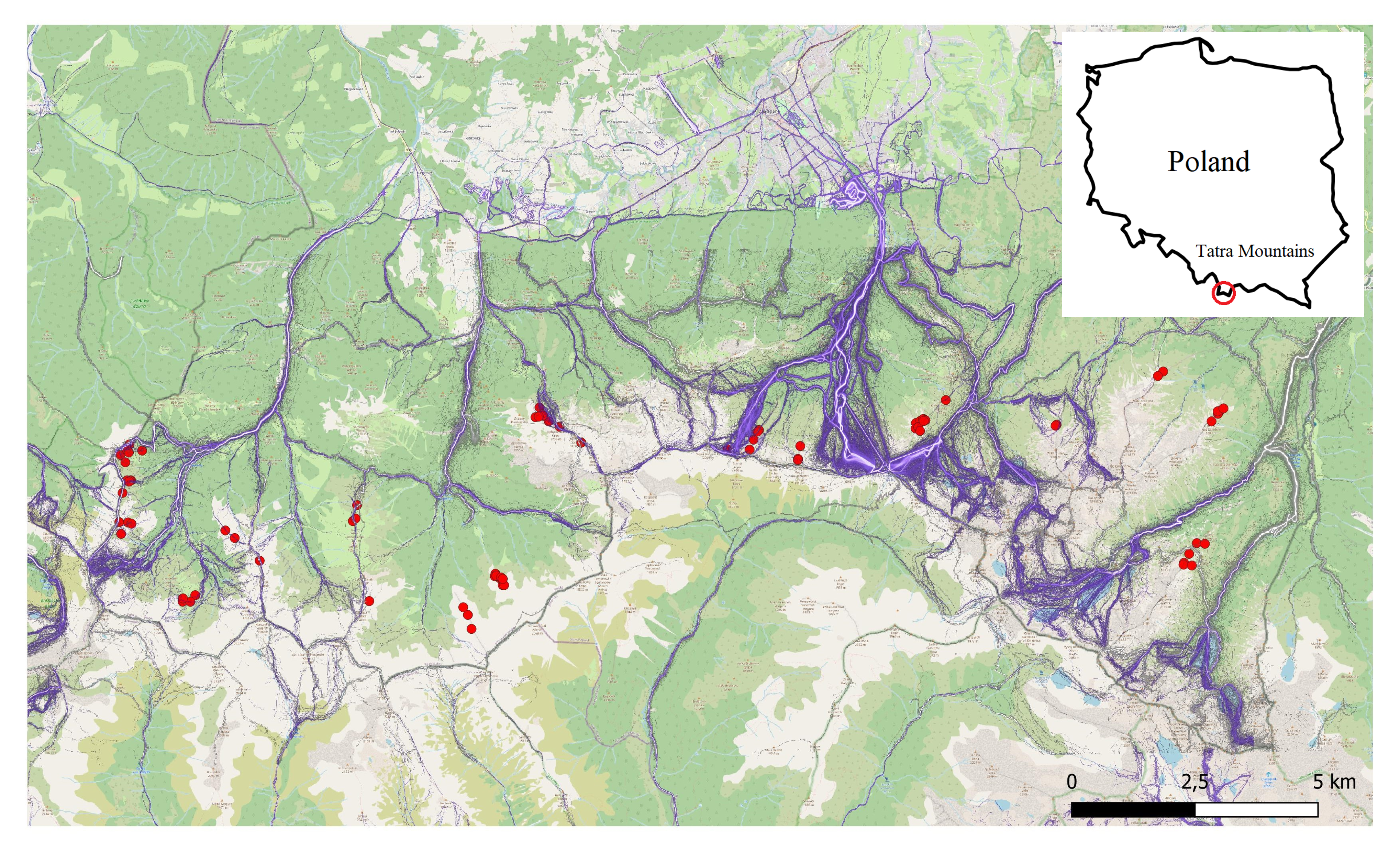

Supplement: Supplementary file 1 — FIGURE A1. Distribution of tourist traffic in Polish Tatra Mountains (purple lines; source: Strava; freemap.sk), and black grouse occurrence (red points). [file ECE3-15-e71231-s002.png]

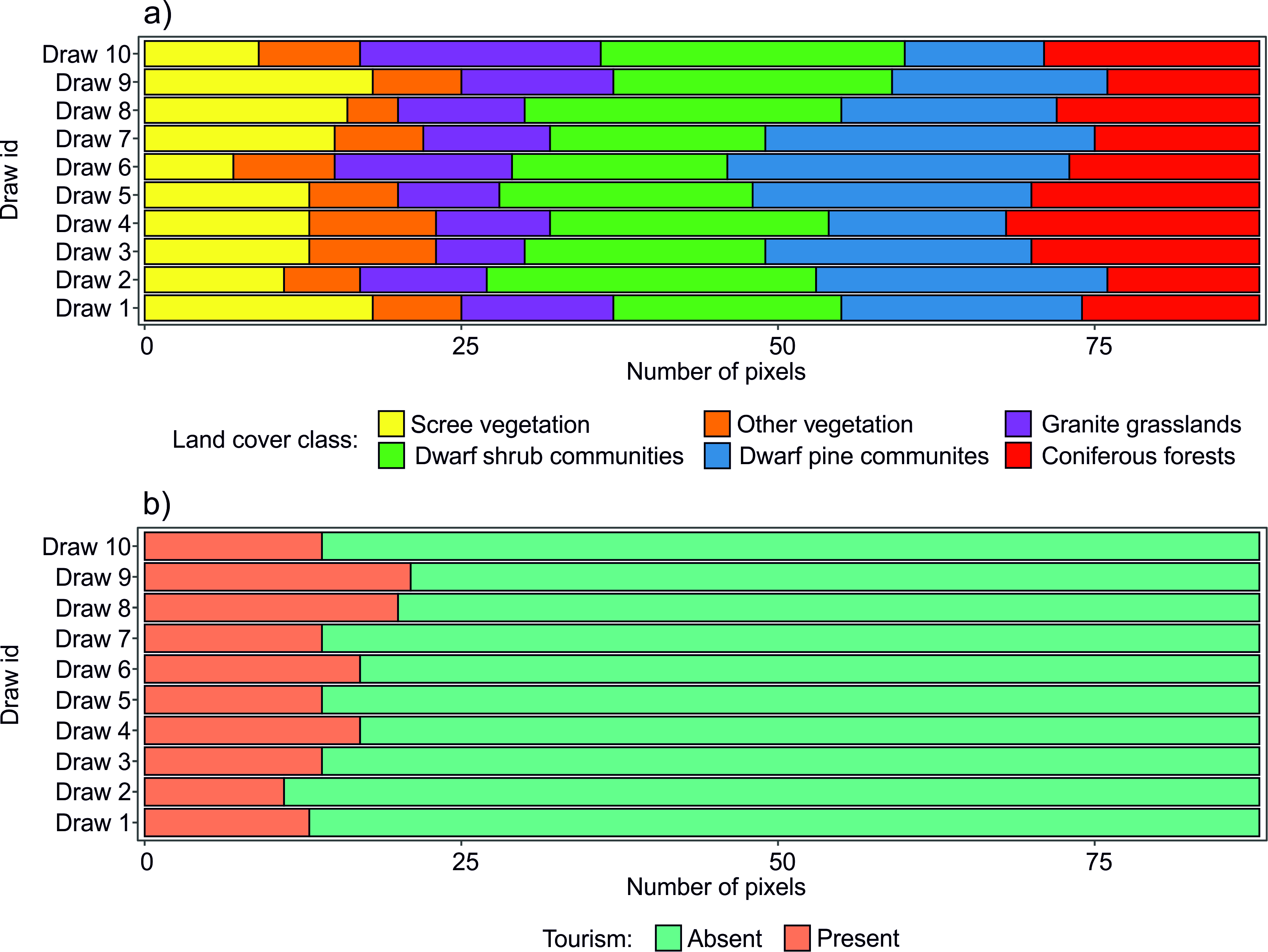

Supplement: Supplementary file 2 — FIGURE A2. A comparison of the affinity of locations representing black grouse’s pseudoabsences to land cover classes (a) and tourism pressure (b) among ten draws of pixels representing pseudoabsences of black grouse. [file ECE3-15-e71231-s004.jpg]

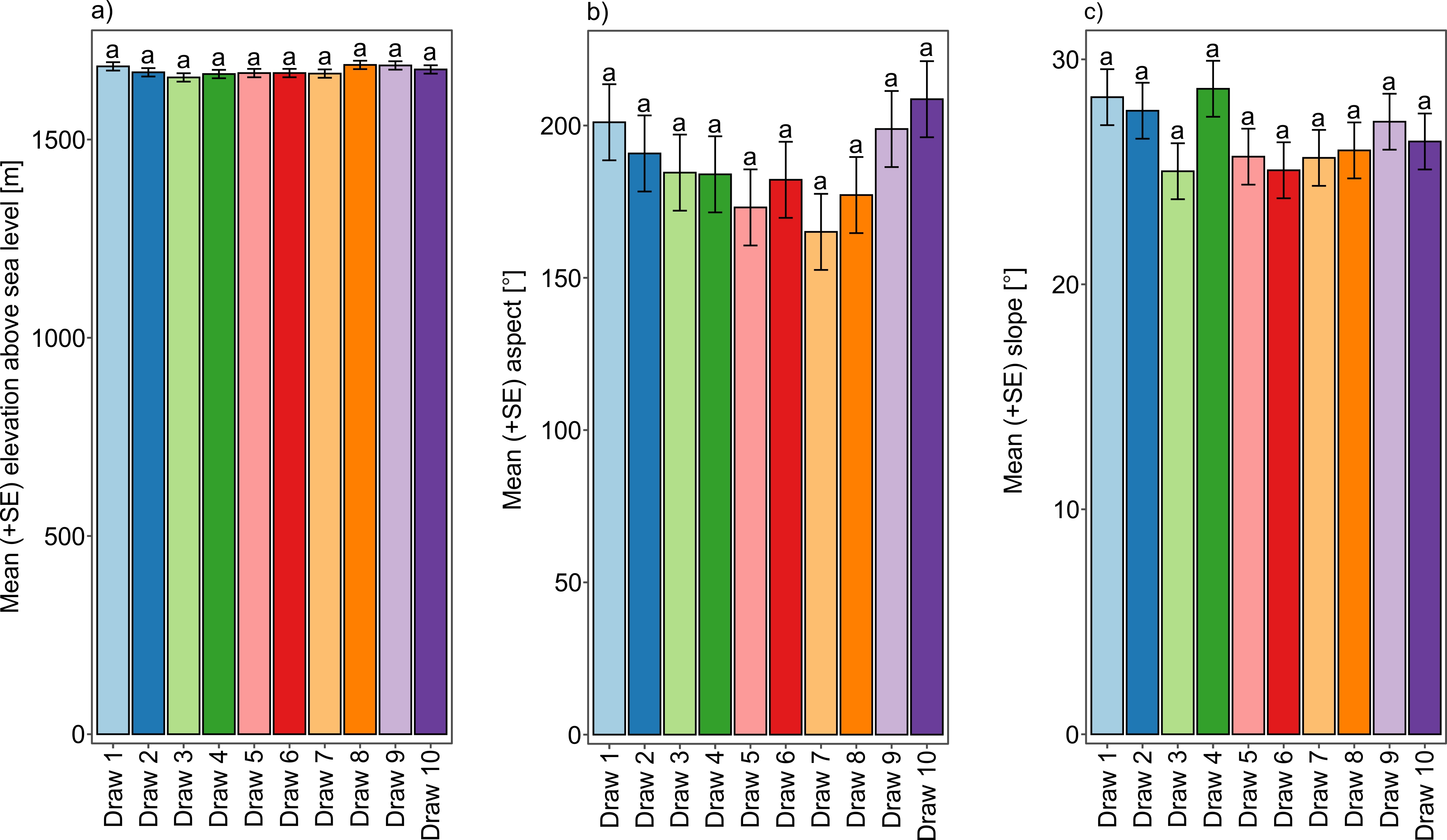

Supplement: Supplementary file 3 — FIGURE A3 Differences in mean (+SE) elevation above sea level (a), aspect (b), and slope (c) among ten draws of pixels representing pseudoabsences of black grouse. Comparisons were based on ANOVAs and Tukey’s posteriori tests. Letters show the significance of results from the post hoc Tukey’s posteriori test. Groups marked by the same letter do not differ significantly at p = 0.05. For model parameters see Table A3. [file ECE3-15-e71231-s001.jpg]
