# Supplementary material for: To Understand the Elusive: How to Avoid the Disappearance of the Black Grouse at the Edge of Its Continuous Range?
Source: Ecol Evol. 2025 Apr 23;15(4):e71231. doi: 10.1002/ece3.71231 (PMC12015741; doi:10.1002/ece3.71231)
Supplement: Supplementary file 4 — Appendix S1. [file ECE3-15-e71231-s003.docx]

**Appendix**

**Table A1.** Comparison of AICc of all GLMs (models with all possible combinations of predictors) used in the model selection procedure. Plus (+) indicates a presence of categorical predictor in particular GLM. Global GLM is marked in *italics*. Null (intercept-only) GLM is marked in **bold**.

| **(Intercept)** | **Elevation above sea level** | **Aspect** | **Land cover class** | **Tourism pressure** | **Slope** | **df** | **AICc** | **ΔAICc** |
| --- | --- | --- | --- | --- | --- | --- | --- | --- |
| -13.09481 | 0.00919 |  | + |  | -0.10638 | 8 | 183.90343 | 0.00000 |
| -13.28556 | 0.00913 | 0.00150 | + |  | -0.10844 | 9 | 185.30601 | 1.40259 |
| -12.18302 | 0.00876 |  | + | + | -0.11285 | 9 | 185.59525 | 1.69182 |
| *-12.52962* | *0.00879* | *0.00132* | *+* | *+* | *-0.11331* | *10* | *187.22677* | *3.32335* |
| 2.12413 |  |  | + | + | -0.11242 | 8 | 197.95901 | 14.05558 |
| 1.62395 |  |  | + |  | -0.09658 | 7 | 199.15749 | 15.25407 |
| 1.85628 |  | 0.00121 | + | + | -0.11241 | 9 | 199.59997 | 15.69654 |
| 1.30514 |  | 0.00167 | + |  | -0.09841 | 8 | 200.18943 | 16.28601 |
| -8.68613 | 0.00636 |  |  |  | -0.08393 | 3 | 209.94213 | 26.03871 |
| -7.88120 | 0.00601 |  |  | + | -0.08919 | 4 | 211.37550 | 27.47208 |
| -8.69988 | 0.00634 | 0.00035 |  |  | -0.08423 | 4 | 211.97301 | 28.06959 |
| -7.90667 | 0.00600 | 0.00021 |  | + | -0.08924 | 5 | 213.47267 | 29.56924 |
| -14.70432 | 0.00860 |  | + | + |  | 8 | 214.11933 | 30.21591 |
| -12.86756 | 0.00754 |  | + |  |  | 7 | 214.46444 | 30.56101 |
| -14.91049 | 0.00859 | 0.00101 | + | + |  | 9 | 215.89503 | 31.99160 |
| -12.91620 | 0.00749 | 0.00063 | + |  |  | 8 | 216.47860 | 32.57518 |
| 2.22328 |  |  |  | + | -0.08533 | 3 | 220.96871 | 37.06529 |
| 1.78053 |  |  |  |  | -0.07353 | 2 | 221.95973 | 38.05630 |
| 2.14360 |  | 0.00042 |  | + | -0.08546 | 4 | 222.96907 | 39.06565 |
| 1.66188 |  | 0.00072 |  |  | -0.07424 | 3 | 223.74643 | 39.84300 |
| -0.59784 |  |  | + |  |  | 6 | 227.40096 | 43.49753 |
| -0.79678 |  | 0.00096 | + |  |  | 7 | 229.10694 | 45.20351 |
| -0.62092 |  |  | + | + |  | 7 | 229.45692 | 45.55349 |
| -0.84695 |  | 0.00105 | + | + |  | 8 | 231.10548 | 47.20205 |
| -7.67428 | 0.00455 |  |  |  |  | 2 | 239.11424 | 55.21081 |
| -8.90426 | 0.00521 |  |  | + |  | 3 | 239.44404 | 55.54061 |
| -7.67203 | 0.00457 | -0.0002 |  |  |  | 3 | 241.15397 | 57.25055 |
| -8.90456 | 0.00521 | <0.00001 |  | + |  | 4 | 241.53842 | 57.63499 |
| **<0.00001** |  |  |  |  |  | **1** | **246.01080** | **62.10737** |
| -0.02857 |  |  |  | + |  | 2 | 247.91743 | 64.01401 |
| -0.01961 |  | 0.00011 |  |  |  | 2 | 248.05000 | 64.14658 |
| -0.06349 |  | 0.00018 |  | + |  | 3 | 249.96743 | 66.06400 |

**Table A2.** Mean values for: altitude, slope and aspect with corresponding 95% confidence interval.

* 95% confidence interval for mean.

| **Pseudoabsences** | | | | | | | | | | |
| --- | --- | --- | --- | --- | --- | --- | --- | --- | --- | --- |
| **id** | **Altitude** | +/ **95CI*** | **Altitude max** | **Altitude min** | **Slope** | +/- **95CI** | **Slope max** | **Slope min** | **Aspect** | **+/- 95CI** |
| 1 | 1684,55 | 20,32 | 1864,3 | 1521,9 | 28,3 | 2,7 | 50,0 | 1,3 | 342,1 | 24,8 |
| 2 | 1669,59 | 20,51 | 1864,9 | 1509,6 | 27,7 | 2,5 | 60,2 | 0,1 | 2,9 | 24,8 |
| 3 | 1656,64 | 20,40 | 1862,8 | 1509,3 | 25,0 | 2,4 | 46,9 | 0,0 | 357,9 | 24,0 |
| 4 | 1665,23 | 22,38 | 1868,1 | 1509,7 | 28,7 | 2,8 | 56,7 | 0,0 | 6,5 | 25,4 |
| 5 | 1667,64 | 21,66 | 1867,1 | 1513,5 | 25,7 | 2,2 | 46,5 | 0,0 | 5,8 | 27,2 |
| 6 | 1667,68 | 20,78 | 1857,2 | 1511,4 | 25,1 | 2,4 | 49,0 | 0,0 | 355,4 | 23,3 |
| 7 | 1666,35 | 20,30 | 1859,5 | 1511,7 | 25,6 | 2,5 | 50,4 | 0,0 | 9,0 | 24,3 |
| 8 | 1688,19 | 21,49 | 1864,4 | 1517,2 | 26,0 | 2,7 | 58,1 | 0,0 | 11,6 | 25,2 |
| 9 | 1686,88 | 20,42 | 1861,4 | 1513,9 | 27,2 | 2,4 | 52,3 | 0,0 | 337,0 | 24,5 |
| 10 | 1676,77 | 22,14 | 1861,8 | 1522,5 | 26,3 | 2,3 | 55,7 | 0,0 | 323,4 | 23,8 |
|  |  |  |  |  |  |  |  |  |  |  |
|  |  |  |  |  |  |  |  |  |  |  |
|  |  |  |  |  |  |  |  |  |  |  |
| **Black grouse records** | | | | | | | | | | |
| 1 | 1710,7 | 19,7 | 1869,0 | 1503,5 | 20,0 | 1,7 | 38,7 | 4,1 | 19,4 | 25,8 |
|  |  |  |  |  |  |  |  |  |  |  |
|  |  |  |  |  |  |  |  |  |  |  |

**Table A3**. Parameters of linear models (evaluated by ANOVA analysis) testing for differences in elevation above sea level, slope, and aspect amongst ten draws of pixels representing pseudoabsences of black grouse. Statistically significant results are in bold.

| Response variable | df | *F* | *P* | *R^2^* |
| --- | --- | --- | --- | --- |
| Elevation above sea level | 9 | 1.003 | 0.435 | <0.001 |
| Slope | 9 | 1.154 | 0.322 | 0.001 |
| Aspect | 9 | 1.159 | 0.318 | 0.001 |

**Appendix**

**Fig. A1.** Distribution of tourist traffic in Polish Tatra Mountains (purple lines; source: Strava; freemap.sk), and black grouse occurrence (red points).

**Fig. A2.** A comparison of the affinity of locations representing black grouse’s pseudoabsences to land cover classes (a) and tourism pressure (b) amongst ten draws of pixels representing pseudoabsences of black grouse.

**Fig. A3.** Differences in mean (+SE) elevation above sea level (a), aspect (b), and slope (c) amongst ten draws of pixels representing pseudoabsences of black grouse. Comparisons were based on ANOVAs and Tukey's posteriori tests. Letters show the significance of results from the post hoc Tukey's posteriori test. Groups marked by the same letter do not differ significantly at *P*=0.05. For model parameters see **Table A3**.
